# Expression and sub-cellular localization of an epigenetic regulator, co-activator arginine methyltransferase 1 (CARM1), is associated with specific breast cancer subtypes and ethnicity

**DOI:** 10.1186/1476-4598-12-40

**Published:** 2013-05-10

**Authors:** Melissa B Davis, Xinyu Liu, Shiyao Wang, Jaxk Reeves, Andrey Khramtsov, Dezheng Huo, Olufunmilayo I Olopade

**Affiliations:** 1 The Institute for Genomics and Systems Biology, University of Chicago Biological Sciences Division, Chicago, IL, USA; 2 Department of Human Genetics, University of Chicago Biological Sciences Division, Chicago, IL, USA; 3 Department of Medicine, Center for Clinical Cancer Genetics and Global Health, University of Chicago Medicine, Chicago, IL 60637, USA; 4 Department of Health Studies, Center for Clinical Cancer Genetics, University of Chicago, Chicago, IL 60637, USA; 5 Georgia Health Sciences University, Athens, GA 30602, USA; 6 University of Georgia Medical Partnership, Athens, GA 30602, USA; 7 Department of Genetics, University of Georgia, Athens, GA 30602, USA; 8 Department of Statistics, University of Georgia, Athens, GA 30602, USA

**Keywords:** Tissue-microarray, Breast cancer, Molecular subtypes, CARM1, Epigenetic regulator, Subcellular localization, Ethnic disparities

## Abstract

**Background:**

Co-Activator Arginine Methyltransferase 1(CARM1) is an Estrogen Receptor (ER) cofactor that remodels chromatin for gene regulation via methylation of Histone3. We investigated CARM1 levels and localization across breast cancer tumors in a cohort of patients of either European or African ancestry.

**Methods:**

We analyzed CARM1 levels using tissue microarrays with over 800 histological samples from 549 female cancer patients from the US and Nigeria, Africa. We assessed associations between CARM1 expression localized to the nucleus and cytoplasm for 11 distinct variables, including; ER status, Progesterone Receptor status, molecular subtypes, ethnicity, HER2+ status, other clinical variables and survival.

**Results:**

We found that levels of cytoplasmic CARM1 are distinct among tumor sub-types and increased levels are associated with ER-negative (ER-) status. Higher nuclear CARM1 levels are associated with HER2 receptor status. EGFR expression also correlates with localization of CARM1 into the cytoplasm. This suggests there are distinct functions of CARM1 among molecular tumor types. Our data reveals a basal-like subtype association with CARM1, possibly due to expression of Epidermal Growth Factor Receptor (EGFR). Lastly, increased cytoplasmic CARM1, relative to nuclear levels, appear to be associated with self-identified African ethnicity and this result is being further investigated using quantified genetic ancestry measures.

**Conclusions:**

Although it is known to be an ER cofactor in breast cancer, CARM1 expression levels are independent of ER. CARM1 has distinct functions among molecular subtypes, as is indicative of its sub-cellular localization and it may function in subtype etiology. These sub-cellular localization patterns, indicate a novel role beyond its ER cofactor function in breast cancer. Differential localization among ethnic groups may be due to ancestry-specific polymorphisms which alter the gene product.

## Background

Breast cancer is still one of the leading causes of death among women. It is a complex disease with a wide range of penetrance and expressivity that ultimately results in variable survival rates among patient groupings, including ethnicity groups [1-3]. Incidence and mortality rates are well known to be significantly divergent among women of certain ethnic groups [1,4-6]. In the US, White women traditionally have a higher incidence of the disease overall, but in the pre-menopausal category, women of African descent have the highest incidence rate [1-3,6,7]. Aside from DNA changes, epigenetic modifications, which regulate the expression *potential* of a gene, have been increasingly implicated in breast tumorgenesis [8-13]. However, not much is known about the epigenetic regulators of molecular pathways leading to derivation of specific tumor subtypes.

The multiple subtypes of breast cancer can be considered distinct diseases, given the discrete molecular signatures and divergent clinical outcomes associated with each [14,15]. Molecular subtypes of breast cancer include; Luminal A/B, HER2+, triple negative and basal-like. These subtypes are characterized by the expression of specific hormone receptors and epithelial markers. Explicitly, the luminal subtypes each express ER and/or PR, the HER2+ subtype harbors gene amplification of the ERBB2 gene (more commonly known as HER2) and the triple negative subtypes lack expression of ER, PR and HER2 amplification. The basal-like subcategory of triple negative tumors [16,17] expresses EGFR and ck5/6 and is the most aggressive sub-type, with a higher mitotic index and reduced survival rates.

Interestingly, several epidemiological studies show that breast cancer in African-American (Afr. Am.) women, as compared to European-American (EA) women, are more likely to be Estrogen Receptor negative (40% vs. 25%), Progesterone Receptor Negative (50% vs. 35%), HER2/Neu negative and ‘basal-like’ (44% more likely) [18-20]. International studies that address breast cancer rates and phenotypes in African populations show that premenopausal breast cancer incidence and more aggressive tumor subtypes at are prevalent in these populations [21-23] as well. These reports support a hypothesis that molecular differences, due to the genetic ancestral variation, among ethnic groups are important factors cultivating a unique burden of specific subtypes within certain populations.

Site-targeted chromatin remodeling is another emerging mechanism of tumor etiology. Specifically, histone modifications have been repeatedly implicated in the differential regulation of genes which impact tumorgenesis [18-20,24] including the regulation of steroid and growth signal target genes [25-27]. Recently, studies show that epigenetic haplotypes are associated with ER-negative breast cancer subtypes [28]. One implicated epigenetic regulator is Co-activator-Associated Arginine Methyltransferase 1 (CARM1). It is a chromatin remodeling regulator of steroid hormone signaling pathways, acting through methylation of several proteins [21,22,29], including Histone 3 [23], and has been associated with breast and prostate cancer etiology [18,30-32].

CARM1 is a member of the Protein Arginine Methyltransferase (PRMT) family and was first implicated in steroid receptor signaling through its interaction with the nuclear receptor p160 co-activators SRC-1 and GRIP1 [33]. In breast cancer, CARM1 has been recently shown to regulate estrogen dependent cell proliferation through upregulation of the transcription factor E2F1, an essential component of cell cycle regulation [34,35]. In addition to histone methylation, CARM1 has been implicated in methylation of other proteins, including SRC-3 which indirectly impacts estrogen mediated breast cancer cell line proliferation [36]. Immunoprecipitation of CARM1, in complex with p53, CBP, Sp1 and cJun, at the ER promoter locus [37] implicates CARM1 as a regulator of ER expression. In fact, CARM1 is required for ER dependent breast cancer cell differentiation [38,39] and this suggests CARM1 may impact the ER status in breast cancer.

To determine if CARM1 might be involved in the development of specific breast tumor subtypes we have measured the CARM1 expression levels, in adjacent-normal and tumor tissues from a cohort of over 500 breast cancer patients from the US (Chicago, IL) and Nigeria, Africa. Here, we report the associations of CARM1 expression and sub-cellular localization across 11 clinical variables, including tumor types, ethnic groups and overall survival.

## Results and discussions

### Sub-cellular localization of CARM1 in breast cancer is molecular subtype dependent

**Figure 1:**
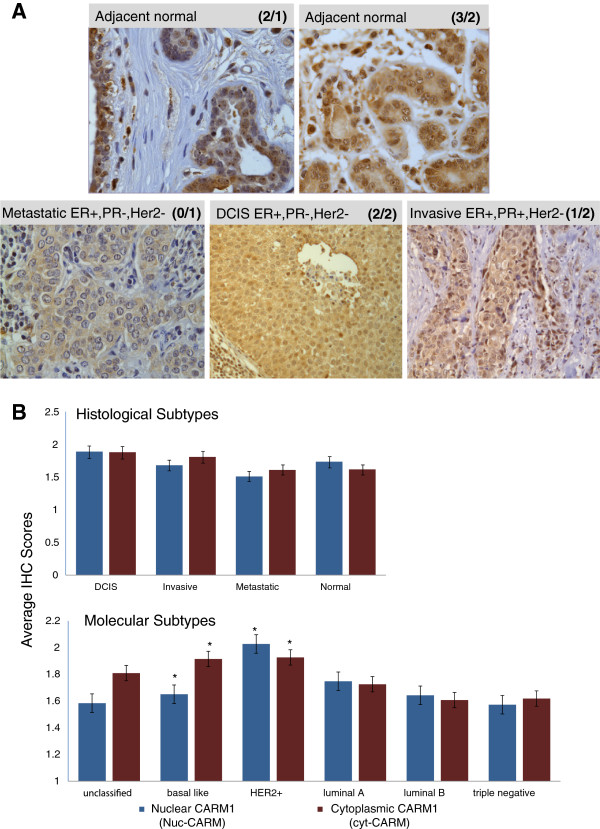
**CARM1 expression and sub-cellular localization of across molecular and histological tumor subtypes. A**. Representative CARM1 IHC stains for Adjacent-normal (top row) and tumor (bottom row) tissue cores. There is a diverse range of both cytoplasmic and nuclear expression levels for each pathological and histological subtype. The specific histological subtype and nuclear receptor status are indicated on respective images. The CARM1 nuclear/cytoplasmic scores are indicated in parentheses (e.g. (3/2) = nuclear score of 3 and cytoplasmic score of 2). **B**. Nuclear and Cytoplasmic CARM1 expression tumor score averages, grouped by histological tumor types (top) and molecular tumor subtypes (bottom). Histological subtypes appear to show very little variation in CARM1 expression, with small variations in localization in the invasive and metastatic tumor categories. Normal tissue indicates CARM1 is usually highest in the nucleus. For the molecular subtypes, there is a wide range of variation among the categories. HER2+ tumors have the highest relative expression of CARM1, while basal-like subtypes have the most divergent localization between nucleus and cytoplasm, with the majority of the protein residing in the cytoplasm, contrary to what is seen in normal tissues overall. Asterisks denote findings that were statistically significant and are outlined in Table 1. Error bars are shown to denote Standard Deviation(1).

CARM1 has previously been shown to function as an epigenetic transcriptional regulator in steroid hormone pathways [25,34]; therefore, we anticipated the majority of the CARM1 protein would be nuclear. With no regard for tissue type or tumor subtypes, based on a Chi-square test of independence (P = 0.001), we found that overall there is a correlation of nuclear and cytoplasmic expression levels of CARM1. Table 1 summarizes the distribution of overlapping nuclear and cytoplasmic scores for all samples analyzed. However, our further investigation has determined that, while correlated, distinct levels of cytoplasmic CARM1 (cyt-CARM1) and nuclear CARM1 (nuc-CARM1) create unique patterns of sub-cellular localization relative to tumor-type categories. Figure 1A shows examples of this variation in expression levels and localization in representative immunostains of adjacent-normal (top row) and tumor (bottom row) samples. When samples are grouped by histological subtypes (Figure 1B – top), the overall average expression of cyt-CARM1 and nuc-CARM1 levels do not appear to be significantly different from each other. However, when samples are grouped by molecular subtypes, significantly different levels of both expression and sub-cellular localization of CARM1 are detected (Analysis of Variance (ANOVA) p<0.0001) (Figure 1B-bottom).

**Table 1:** IHC scores range from 0-3

		**nuc-CARM1 IHC scores**				
		**0**	**1**	**2**	**3**	**TOTAL**
**cyt-CARM1 IHC scores**	**0**	***7***	4	3	0	**14**
	**1**	53	***36***	75	10	**174**
	**2**	19	55	***174***	64	**312**
	**3**	2	9	36	***28***	**75**
	**TOTAL**	**81**	**104**	**288**	**102**	**635**

The bold italics highlights the numbers of cores with identical scoring of nuc-CARM1 and cyt-CARM1, indicating equalized localization. There is a general correlative trend of similar expression levels between nuclear and cytoplasmic localization overall, without regard for tumor subtypes.

As CARM1 has previously been shown to function as an epigenetic transcriptional regulator in steroid hormone pathways [25,34], we expected the majority of IHC staining for CARM1 to be nuclear. However, our observations reveal that while the overall expression levels of nuclear vs. cytoplasmic CARM1 are similar across the sample population, there are specific tumor molecular phenotypes where sub-cellular protein levels are distinct. Accordingly, for the remainder of our investigations we distinguish nuclear from cytoplasmic expression as we investigate associations with our patho-clinical variables. For this purpose, we assigned separate nuclear and cytoplasmic scores for each tissue sample to retain the relative levels of sub-cellular localization. An overall summary of the statistical results for each of the 11 variables tested can be found in Table 2.

**Table 2:** Summary of statistical tests for each of the 11 variables investigated

	**Simple linear regression tests for cyt-CARM1**				**Simple linear regression tests for nuc-CARM1**			
	**N**	**P-value**	**Beta**	**SE(Beta)**	**N**	**P-value**	**Beta**	**SE(Beta)**
CK5/6*	574	***0.0188***	0.099	0.042	573	0.7506	0.0178	0.0559
EGFR*	574	***0.0061***	0.1377	0.0501	573	0.7031	0.0254	0.0667
Tumor grade*	416	***0.0102***	0.128	0.0496	415	0.1030	0.1059	0.0648
	**Cumulative logistic regression tests for cyt-CARM-1**				**Cumulative logistic regression tests for nuc-CARM-1**			
	**N**	**P-value**	**OR**	**95% OR CI**	**N**	**P-value**	**OR**	**95% OR CI**
**ER**	482	0.3053	0.834	0.588 - 1.181	481	0.6592	1.079	0.769 - 1.515
**PR**	480	0.8028	0.954	0.656 - 1.385	479	0.9608	1.009	0.702 - 1.452
HER2**	477	**0.0023**	1.948	1.269 - 2.989	476	**<0.0001**	2.596	1.702 - 3.959
**Age**	443	0.0669	0.989	0.977 - 1.001	629	0.5913	0.997	0.985 - 1.009
**Tumor size**	427	0.252	1.039	0.973 - 1.110	596	0.5936	1.018	0.955 - 1.085
	**One-way ANOVA tests for cyt-CARM-1**				**One1-way ANOVA tests for nuc-CARM-1**			
	**N**	**P-value**	**RMSE**	**Significant differences**	**N**	**P-value**	**RMSE**	**Significant differences**
Molecular subtype**	475	**0.0004**	0.6657	(HER2, Basal) > LumA	474	**0.0004**	0.872	HER2 > (LumA, Basal)
**Histological subtype**	576	**0.0319**	0.69	(none found)	575	0.1049	0.9146	(none found)
Ethnicity**	534	**0.003**	0.7019	Afr > (Cauc, AfrAm)	533	**0.0001**	0.0001	(Cauc, AfrAm) > Afr.

Depending on the structure of the data for each variable, one of three statistical tests were used; Simple Linear Regression, Cumulative Logistic Regression or ANOVA. See methods for details of stats applications. Asterisks and bold font P-values indicate significant associations at 0.05 level. All significant associations are described in detail in the text. *RMSE* root mean squared error; *CI* confidence interval; *SE* standard error; *OR* odds ratio; *E(Y)* expected values of CARM1 (Y) at indicated level of variable’s score (0–3).

### Relationship between CARM1, tumor subtypes and intra-patient tumor progression

Because the relative sub-cellular localization of CARM1 is directly indicative of its biological function and possible influence on tumor progression, we determined whether there were distinct levels of nuc-CARM1 or cyt-CARM1 associated with malignancy or specific histo-pathology tumor types. Specifically, we first investigated whether nuc-CARM1 or cyt-CARM1 expression levels were associated with the histological categories; Adjacent-Normal (normal cells adjacent to tumors), Ductal Carcinoma In Situ (DCIS), Invasive and Metastatic (Lymph Node (LN)) (Figure 1B and Table 2, Histological type). We conducted this analysis without strict concern for increasing severity. While we did not find a significant association between these tumor categories and nuc-CARM1 expression (P=0.1049, n=575) there was a significant association with cyt-CARM1 expression (P=0.0319, n=576). On average, there is a slightly higher level of cyt-CARM1in the DCIS and invasive tumors compared to normal and metastatic tumors. This observation may indicate a tumor specific difference in CARM1 regulation or function linked to the tumor cells’ microenvironment, which may be lost during the Epithelial to Mesenchymal Transition (EMT) of cells as they metastasize to a different site.

**Figure 2:**
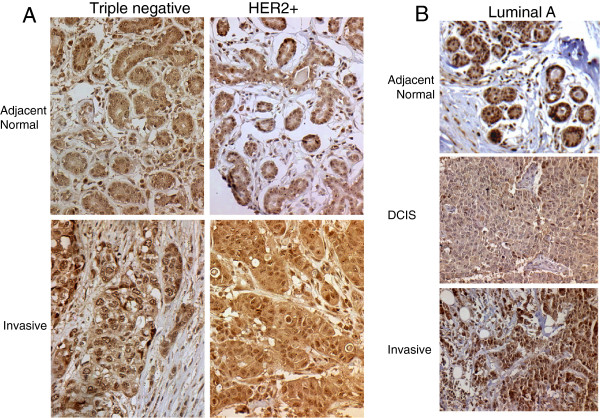
**Inter-patient assessments of CARM1 levels.** Case matched samples are shown for the indicated histo-pathological tumor type (normal, DCIS, invasive or metastatic) and molecular subtypes (Triple negative, HER2+ and Luminal A). Tissues from individual cases are stacked in columns in the order of increasing severity. **A**. Representative stages of progression and the representative differential expression of normal to invasive. We observe high levels of CARM1, even within the adjacent normal, which may indicate pre-cancerous upregulation. **B**. Representative stages of progression from normal to DCIS to Invasive. Again, we note high levels of CARM1 in the adjacent normal; however, we do not always observe increasing expression with severity of pathology. There are significant associations with sub-cellular localization, indicating the CARM1 levels may be higher prior to carcinogenesis but localization changes with function throughout the process.

To more distinctly address the question of CARM1 in association with progression of tumor stages, we investigated whether there were significant differences of CARM1 expression levels along the spectrum of tumor progression within individual patients. There were 222 patients with multiple TMA samples in our cohort. Of these, 178 had samples with distinct histological subtypes. Overall, there was an intra-patient correlation (i.e. a patient who has a high score in an invasive tumor is likely to also have a high score in adjacent normal) Figure 2 shows representative stains of our intra-patient samples. A pairwise comparison of intra-patient histology categories shows significant differences in cyt-CARM1 levels between adjacent-normal and invasive tissue types (WSR P=0.03 n=60). This suggests that from the progression of normal to invasive malignancy the localization of CARM1 becomes ‘more cytoplasmic’, which would imply that the specific function of CARM1 may shift more toward cytoplasmic targets during progression of tumorigenesis. Other pairwise comparisons indicated there was no significant change in CARM1 levels between one pathological stage to the next (Table 3). However, limitations due to our low intra-patient numbers, likely prevented us from finding any additional significant associations. The summary of intra-patient samples is outlined in Additional file 1: Table S1.

**Table 3:** Inter-patient analyses based on Wilcoxon rank sum tests

**Pathology transition**	**Cyt-CARM1**		**Nuc-CARM1**	
	**n**	***P *value**	**n**	***P *value**
**Normal vs DCIS**	16	0.103	16	0.371
Normal vs invasive*	***60***	***0.036***	60	0.684
**DCIS vs invasive**	36	0.727	36	0.558
**Invasive vs metatstatic**	35	0.948	34	0.928
**DCIS vs metastatic**	n.d.	n.d.	n.d.	n.d.

Bold italics indicates statistically significant associations. The only significant finding was between normal and invasive tumor types.

**Figure 3:**
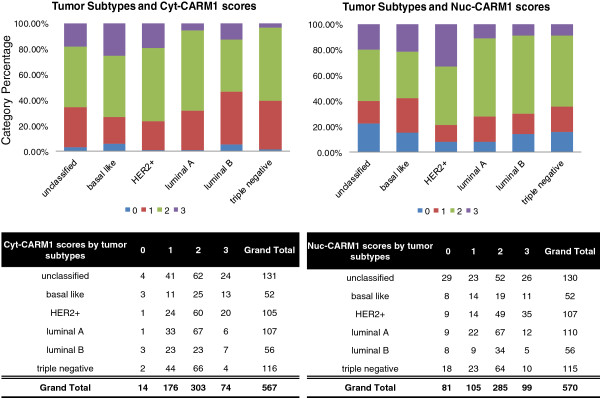
**CARM1 expression score distributions of individual tumors.** Top, bar graph indicating cumulative percentages of each score level within indicated molecular subtypes. Bottom, data table indicates actual numbers of cores, showing distributions of CARM1 scores within each molecular tumor subtype.

To investigate CARM1 expression among molecular tumor subtypes, we first utilized histological markers to sort each tumor sample into the categorical molecular subtypes; Basal-like, Luminal A, Luminal B, HER2+/ER- (HER2+) and “unclassified” [40-44]. In contrast to the histological subtypes, an ANOVA analysis across molecular subtype categories revealed that cyt-CARM1 levels are extremely different across these subtype groupings (P=0.0004, n=475) (Table 2 and Figure 3). Similarly, nuc-CARM1 expression levels also show a significant difference across tumor subtypes (P=0.0004, n=474). Specifically, nuc-CARM1 IHC scores reveal a higher level of CARM1 in the nuclei of HER2+ tumor cells compared to Basal-like and Luminal tumor cells while cyt-CARM1 IHC scores reveal higher levels of CARM1 in the cytoplasm of both Basal-like tumor cells and HER2+ subtypes (Figure 3) compared to the other molecular subtypes. HER2+ tumor had the highest expression of CARM1 overall.

### Relationship between CARM1 and steroid nuclear receptor (ER and PR) status

Because the expression of specific nuclear receptors is correlated to molecular subtype categories, we have investigated the direct correlation of CARM1 with steroid receptors ER and PR. Using cumulative logistic regression (CLR), we determined whether there were any significant associations among ER or PR status and CARM1. We found that in a tumor subtype independent test there was no nuc-CARM1 or cyt-CARM1 association with ER (CLR P=0.6592 and 0.3053, respectively) or PR (CLR P=0.8028 and 0.9608, respectively). This finding is very intriguing as CARM1 has been shown to function as an ER cofactor; however, our study did not detect a direct correlation between ER status and CARM1 expression. In terms of sub-cellular localization, there was a noticeable trend of relatively more nuclear localization of CARM1 in the ER-positive tumor categories and more cytoplasmic localization of CARM1 in ER-negative tumors; however, this trend was not statistically significant (WSR P= 0.062).

In an ER-negative context, the ‘typical’ mode of hormone signal target-gene regulation can become atypical in order to achieve hormone independent proliferation and evasion of apoptosis. CARM1 works in concert with other transcription cofactors in order to mediate estrogen target gene regulation [45,46]; however, its similar function in the androgen pathway [28,30,47] in prostate cells has been shown to gain independence from the androgen signal [25,48]. We believe our data may suggest similar steroid independence may be achieved for CARM1 function in breast cancer subtypes that are estrogen independent (ER-negative). Conversely, in the ER positive tumors, where CARM1 would most likely function as an ER nuclear cofactor, the protein is usually localized to the nucleus. This suggests that CARM1 epigenetic transcriptional function may become limited, removed or at the very least, altered, in the absence of the estrogen steroid receptor. In addition, given the link of CARM1 to the regulation of ER gene expression, perhaps the lack of CARM1 in the nuclei of ER-negative tumor cells is a cause for the ER-negative status and this hypothesis should be investigated further.

### Relationship between CARM1 and growth factor receptors, HER2 and EGFR

**Figure 4:**
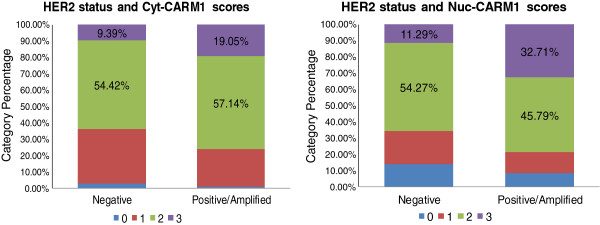
**CARM1 localization is associated with HER2 status.** Bar graphs are shown depicting the distributions of individual CARM1 scores for all tumors stratified by HER2 status.

The significant differences in cytoplasmic and nuclear levels of CARM1 between the basal-like and luminal tumor subtypes, suggests that hormone receptors, other than ER, interact with CARM1. Specifically, the “basal-like” and “unclassified” categories lack ER expression and have CARM1 predominantly localized to the cytoplasm. A cumulative logistic regression analysis revealed a significant association between HER2 status and both cyt-CARM1 and nuc-CARM1 (Figure 4). Specifically, we found a positive association with HER2 status and cyt-CARM1 (P=0.0023, Odds Ratio (OR) = 1.95 95% CI: 1.27-2.99). We also found a positive association between HER2 status and nuc-CARM1 (WSR P=0.002) (Figure 4). Similarly, the average expression trends among the tumor subtype groups, shows that HER2+ tumors have a relatively higher level of both nuclear and cytoplasmic expression when compared to averages in the other tumor types (Figure 1B). In addition, distributions of CARM1 scores among the molecular subtypes indicate that the HER2+ tumors have the largest percentage of the highest nuc-CARM1 scores (Figure 3). These data indicate that tumors with an amplified HER2 gene also have high levels of CARM1 expression overall. Also, these data show that CARM1 is preferentially localized to the nucleus in the presence of high levels of HER2.

In addition, for the Epidermal Growth Factor Receptor (EGFR), we found a positive association with cyt-CARM1 (P=0.0061) but no significant association with nuc-CARM1 (Table 2). This indicates EGFR expression correlates with higher levels of CARM1 expression, but only when CARM1 is preferentially localized to the cytoplasm. This observation might suggest that CARM1 could play a functional role in the cytoplasm, possibly through some interaction with EGFR, as it too is preferentially localized to the cytoplasm. In addition, because the only molecular subtype that expresses EGFR is the basal-like tumor, our observations may give insight to the etiology of the basal-like subtype.

**Figure 5:**
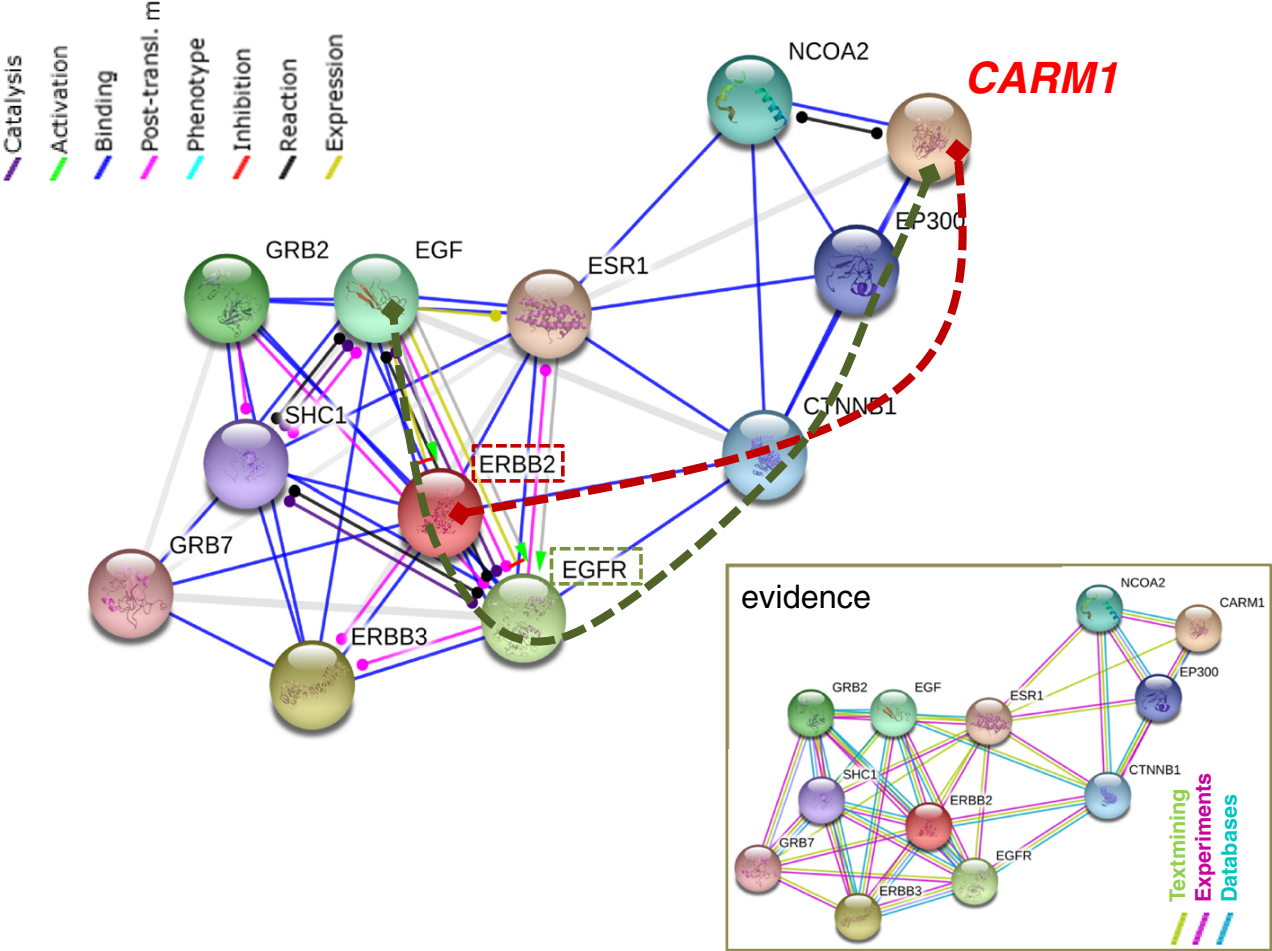
**Model of CARM1 interaction with HER2 and EGFR.** A STRING database interaction network is modified to indicate putative interaction paths between CARM1 to HER2 and CARM1 to EGFR. Dashed red line highlights a potential physical connection through protein-protein binding of CARM1 in complex with HER2. The dashed green line indicates a potential functional connection to EGF through both post-translational modifications and physical interaction with EGFR complexes. In an ER-negative tumor, the ESR1 interaction is absent; therefore the interaction would occur through CTNNB1. The evidence box shows the source and type of data used to designate the protein interactions.

To model these potential interactions, we have identified two independent lines of evidence that indicate HER2 protein may physically interact with CARM1protein in the HER2+ tumors, while EGFR may physically or functionally interaction with CARM1 protein in basal-like tumors. This model is summarized in Figure 5, where we show a modified STRING Database network [49] suggesting how CARM1 may physically interact with HER2 using protein-protein binding data. In addition, we show a possible connection to the EGF pathway through protein-protein binding data with EGFR. These pathways could presumably circumvent the usual CARM1-ER protein-protein interaction, effectively expanding CARM1 function beyond its role as an ER cofactor in breast tissue.

Previous studies support these models by showing that CARM1 function is not isolated to chromatin associated histones [50]. We hypothesize that cytoplasmic methylation targets are modified more often within basal-like tumor subtypes where cyt-CARM1 is higher. In fact, a recent study shows evidence that EGFR, a receptor which is exclusively expressed in basal-like subtypes, is methylated by PRMT5, a member of the CARM1 protein family [51]. Their model speculates that this EGFR arginine methylation might enhance the protein’s autophosphorylation and attenuation of EGFR-mediated ERK activation. If EGFR is a CARM1 target, it would only occur in basal-like tumor types as other subtypes do not express EGFR. This lends significant insight to the etiological mechanisms that may drive aggressive progression of this particular molecular subtype. However, more evidence is needed to determine if CARM1 would target the same arginine and also to determine the functional consequences of such a modification.

### Relationship between CARM1 and other clinical characteristics (age, stage, grade, size)

Using a cumulative logistic regression we investigated associations of CARM1 with age at disease onset. We detect a trend of younger women to have higher levels of cyt-CARM1; however the effect was not large enough to be significant (P= 0.06).

In addition, we utilized simple linear regressions and ANOVA analyses to investigate the association of CARM1 with other clinical annotations; including tumor size, grade and stage. There was an expected significant difference in tumor sizes among ethnic groups (WSR P= <0.0001) which matches the national trends. Specifically, the mean tumor size was 4.65 cm for the African ethnic group, (range of 1.5 cm -16 cm) and 3.33 cm for the domestic group (which includes Af. Am. and Caucasian patients) with a range of 0.2 cm to 17 cm. However, we did not find a significant trend or association between tumor size and cyt-CARM1 or nuc-CARM1 (P=0.252 and 0.5936 respectively).

Similarly, we did find a positive trend between nuc-CARM1 and tumor grade, but this was not significant (P=0.103). Interestingly, there was a significant positive association between cyt-CARM1 and tumor grade (P=0.010). This indicates that the increased localization of CARM1 to the cytoplasm corresponds to the increased aggressiveness of the tumor, suggesting CARM1’s cytoplasmic function may be involved with increased cellular proliferation and/or invasiveness.

### Overall comparative relevance of our cohort findings as relates to ethnic disparities

**Figure 6:**
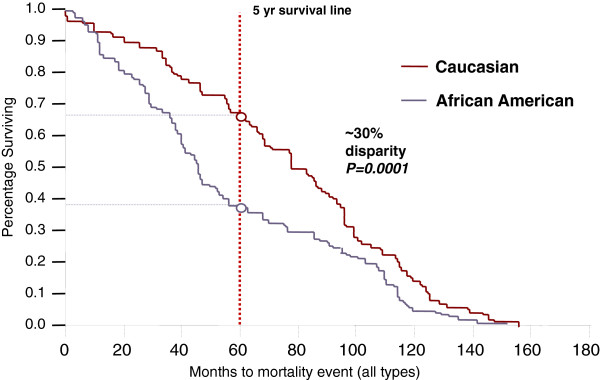
**Ethnic disparities in survival rates.** A Kaplan-Meir Analysis indicates our cohort suffers from the typical disparities in ethnic survival trends between Caucasian and African American (Afr. Am.) patients. Specifically at the 5-year survival mark, there is a 30% gap between the two groups.

Many of our CARM1 associations correlate with tumor categories that are reported to be more prevalent in specific ethnic groups. For instance, there is an association of cyt-CARM1 in basal-like tumors, which is a tumor subtype that has relatively higher incidence rates in certain ethnic groups. Accordingly, we wanted to investigate whether CARM1 expression has associations with ethnicity. However, to ensure our sample set is appropriate for this testing, we first determined if our cohort is representative of current trends in ethnic group disparities, nationally. To test, within our cohort, the national relevance of ethnicity associations commonly identified in other domestic cohorts, we first investigated the well-documented general cancer disparities observed between Afr. Am. and Cau women. We determined concordance with national findings and confirmed a 30% disparity in 5-year-survival (Figure 6). Overall, this is consistent with the national trend [52] (p<0.0001) showing a higher mortality rate in the Afr. Am. group (Figure 6).

Also, we determined whether our cohort has sufficient power to detect typical survival disparities based on commonly used molecular makers. Specifically, we stratified our population based upon the three most significant histological markers known to predict survival trends; ER, PR and HER2. We investigated ER status disparities and observed a significant association of ER status among ethnic groups in our cohort (Chi-Square P>0.0001), with Cau having more ER positive and Afr.Am. and Afr having more ER negative tumors. Additionally, there was a significant ethnicity differential in tumor grade (WSR P=0.046), PR status (P<0.0001) and tumor subtypes as defined as Basal-like, Luminal A, Luminal B, HER2+/ER- and ‘unclassified’ (P=0.001). In addition, we observed these molecular markers show significant survival associations (Wilcoxon p<0.0001 for each), all in concordance with national trends. These findings indicate our patient subset for survival analysis is appropriate for detecting survival disparities linked to molecular markers in clinical practice.

### Ethnicity findings and potential issues with self-Identified race as a proxy in cancer studies

**Figure 7:**
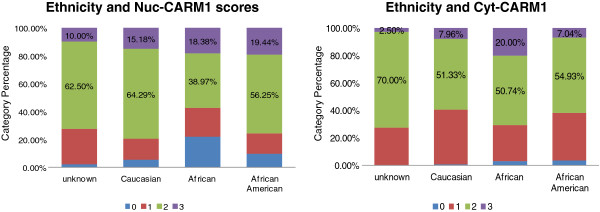
**CARM1 localization is associated with ethnicity categories.** Bar graphs are shown depicting the distributions of individual CARM1 scores for all tumors stratified by self-identified ethnicity.

Because our preliminary analyses demonstrate the similarity of national trends within our cohort, we moved forward with our investigation of associations between CARM1 and ethnicity. Specifically, our cohort was categorized into 3 ethnic groups, African (Afr.), African American (Afr. Am.) or Caucasian (Cau). An ANOVA across a total of 534 samples revealed an overall significant difference in both cyt-CARM1 and nuc-CARM1 expression among the ethnicity groups (Table 2 and Figure 7). Interestingly, our findings indicate that differential nuc-CARM1 expression is more significant than differential cyt-CARM1 (P= 0.0001 and 0.003, respectively). Specifically, pairwise ethnic group comparisons indicate that levels of cyt-CARM1 are higher in Africans than both Afr. Am. (WSR P=0.0013) and Cau (WSR P= 0.010). Correlatively, the levels of nuc-CARM1 expression are significantly higher in Afr. Am. and Cau groups when compared to Africans.

Intriguingly, our findings show that the largest difference in CARM1 expression exists between the two ethnic groups that presumably share relatively more common ancestral genetic background. However, our cumulative evidence may actually indicate that CARM1 expression levels are not correlated with African ancestry but rather indicate a distinct expression pattern specific to our African patient population. Accordingly, we tested whether the CARM1 expression differences associated with ethnicity could be explained by geographical regions. For this analysis we grouped the populations by domestic (Afr. Am and Cau combined) or African sample origin and found significant differences (WSR P=0.0165), though not as significant as our previous analysis.

Our inclusion of native African patients presumably assists with increasing power for comparative ethnicity analyses. Essentially, we have included the African ancestral group as well as the admixed Cau group for our analyses of the admixed Afr. Am. groups. In this manner, we considered ethnicity as an ordered categorical variable of “African-ness” for our drawn conclusions. Cumulatively, our findings suggest that there is a regional bias in CARM1 and that the Afr. Am. group correlates more with its assumed admixed population than with its implied ancestral population.

However, it is possible that the ancestral link for a significant portion of our Afr. Am patients may be in African populations outside of Nigeria. This alternate African ancestry scenario would confound the Nigerian ancestry of Afr. Am. patients in our analysis and could possibly give rise to non-correlation between the Afr and Afr. Am. samples. Accordingly, we should consider our ethnicity findings as preliminary until validation studies can be completed which utilize molecular ancestry measures (See Conclusions, below). Regardless of the analytical limitations in our self-identified admixed groups there is a clear distinction of CARM1 expression and localization in our Afr population.

Ideally, proper identification of ancestry associations with molecular markers requires a method of quantifying ancestry. Whether genetically or epigenetically driven, a biological mechanism of ancestry-specific disease expressivity relies on the premise of a common molecular variant that is stabilized within the descendant group being observed. In the case of CARM1, such a variant must have either ancestral polymorphism origin, which may exist as trans-generational epigenetic imprints mediated by CARM1 [53], or common environmental influences, which could modify or potentiate CARM1 function. There are over 500 known polymorphisms in the CARM-1 gene, of which approximately 10% show unique minor allele frequencies restricted to African ancestral groups in HapMap analyses. Of these, several are non-synonymous changes. Our particular dataset is based upon self-identity and not a quantified measurement of genetic ancestry. Associating self-identified race with a molecular trait can be problematic and especially in consideration of the genetic admixture inherent in our domestic study population of Afr. Am. patients. We are currently conducting follow-up studies to validate the African associations we have detected here. We are using methods of quantifying ancestry and haplotype-block mapping [7,54,55] utilizing such techniques as Ancestral Informative Marker (AIMs) [56,57] genotyping so as to both define individual’s genomic ancestry globally and among CARM1 SNP loci. Specifically, this will allow us to determine the actual CARM1 haplotypes in our population and correlate these with genetic ancestry and CARM1 sub-cellular localization and/or target methylation.

### CARM1 associations with survival

**Figure 8:**
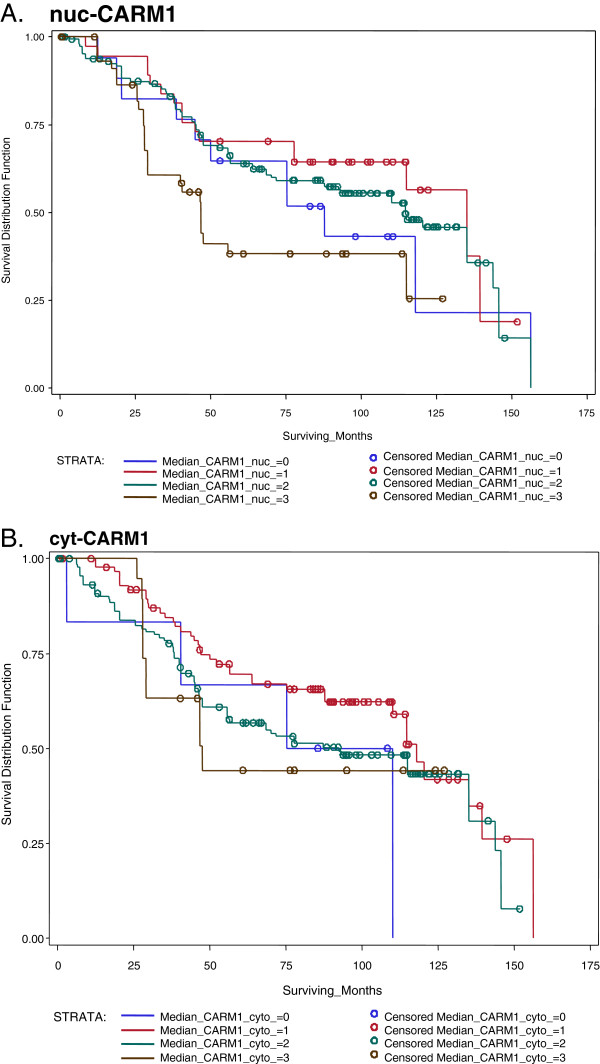
**Localized CARM1 is associated with survival.** We find a slightly significant association with survival and cyt-CARM1, but no association with nuc-CARM **A**. Kaplan-Meier curves for nuc-CARM1. There appears to be an association with CARM1 nuclear expression and survival. **B**. Kaplan-Meier curves for cyt-CARM1 indicate there is no association with CARM1 cytoplasmic expression, at least not with all tumor types considered.

Traditionally, breast cancer survival studies include only the invasive histological subtype; however, we concur with studies that view DCIS as a non-obligate precursor [41] to the invasive tumor, which can be classified into molecular/clinical subtypes with identical markers. Integrating these subtypes into survival analyses lends power to detect factors determining disease progression [41]. Because we did find associations of CARM1 levels in DCIS, we conducted an alternative survival analysis including this histological subtype in our analysis (Figure 8). In doing so, we increased our sample number from 161 to 252, thereby increasing overall statistical power. The median survival time was 9.55 years. (95% CI=I: 3.8-13.9). Our CARM1 survival investigation revealed a marginally significant association of nuc-CARM1 (WSR P=0.0186) but no association with cyt-CARM1 (Table 2 and Figure 8).

**Figure 9:**
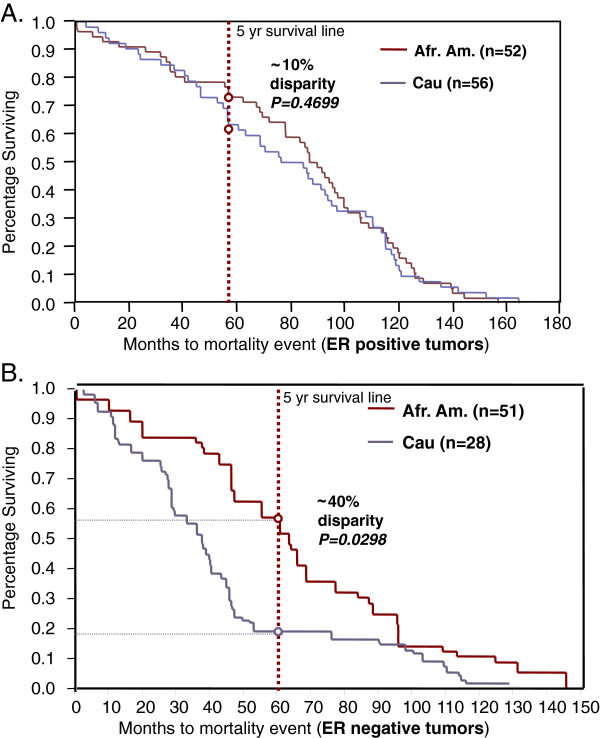
**ER status and subgroup survival trends.** Compared to the 30% disparity in survival between ethnicity groups for all tumor types (Figure 6), disparities decrease to 10% in the ER positive tumor category (top) and increases to 40% (bottom) in the ER negative tumor category. While both ethnic groups show a lower survival rate in ER negative tumor groups, the survival is far worse in the African American group.

Lastly, while the Afr. Am. vs. Cau survival curves mimic the national trends (Figure 6), interestingly, we can detect an increase in mortality disparities within the ER-negative tumor categories (Figure 9), which includes all histological tumor categories. This finding reveals that, first, the ethnic group disparities are not simply due to the ER-negative status and second, that there is a larger ‘race effect’ in the ER-negative sub-categories that was confounded in the ‘all tumors’ survival curve. We hypothesize this reveals a biological difference between these two groups that is nested in specific ER-negative tumor types. It is possible that these survival disparities could be the outcome of a higher number of the more lethal ER-negative tumor subcategory cases in the Afr. Am. category or differences in treatment responses. However, given that the disparities decrease to approximately 10% in the ER-positive tumor categories (Figure 9), and these patients were all recruited and treated from within the same hospital system with standardized treatment protocols; we are focusing our efforts on pathological differences, rather than subtle and subjective patient care variables. Interestingly we did identify an association with CARM1 and survival within the ER-negative tumor types (Additional file 2: Figure S1).

## Conclusions

*We have determined that differential expression and localization of CARM1 is associated with two molecular subtypes of breast cancer (HER2+ and basal-like) but could not identify an association with the histological subtypes or pathological progression of tumors from DCIS to metastasis.* We conclude that the sub-cellular localization of CARM1 is somehow related to the specific differentiation of molecular tumor subtypes. These molecular subtypes are directly linked to the expression of specific hormone receptors. Specifically, we find that the presence of EGFR and HER2 in specific tumor subtypes corresponds with distinct expression and localization of CARM1, suggesting potential interactions between CARM1 and these hormone receptors. Our results show that in tumors where HER2 is over-expressed, CARM1 is generally upregulated with higher levels found in the nucleus. However, when EGFR is expressed (in the absence of ER) CARM1 levels are higher in the cytoplasm. In the absence of HER2 amplification or EGFR, CARM1 has higher levels in the nucleus. Lastly, in the ER- context, CARM1 has higher levels in the cytoplasm. These are insightful findings which cumulatively indicate distinct CARM1 activity and function among specific tumor subtypes. Factors which facilitate the sub-cellular localization CARM1 may play a vital role in breast cancer subtype etiology.

In addition, *we have identified an association between CARM1 expression and ethnicity* in our cohort. Specifically, we observe significantly higher levels of cyt-CARM1 in the African race group, independent of tumor categories, relative to the higher levels of nuc-CARM1 in Afr. Am. and Cau patients. Most tumor sections show an apparent nuclear exclusion of CARM1 in the African samples (data not shown). This suggests an underlying influence on protein localization that may be unique to a specific ethnicity group. This is a plausible hypothesis; given there are polymorphisms in CARM1 which alter the amino acid sequence and are specific to the correlative HapMap ancestral group. Follow-up studies, using Ancestral Informative Markers to quantify genetic ancestry, will help elucidate these ethnicity associations.

## Methods

### Cohort description, sample numbers and objectives

This cohort study was IRB approved at the University of Chicago through the Center for Interdisciplinary Health Disparities Research. Of the 796 patient samples, 160 did not have enough tissue remaining for either nuc-CARM1 or cyt-CARM1 due to previous sectioning of the TMA block. Therefore, our analyses are based on immunohistochemical (IHC) results obtained from 635 samples across 8 tissue-microarrays (TMAs) were joint nuc-CARM1 and cyt-CARM1 could be obtained. Of the total number of complete CARM1 samples, 480 have relevant ER, PR, HER2, c/k 5/6 and EGFR data for the molecular subtype analyses. The TMA sample compilations were designed to investigate the differential expression and statistical associations of oncogenes in breast tumor and adjacent normal tissues. The original complete cohort includes approximately 800 tissue core samples from 549 individuals belonging to African (Nigerian) (308), Afr. Am. (114), Caucasian (Cau) (95) and Native American (1) self-identified ethnic groups (31 patients had no ethnic group information). Domestic samples were obtained from patients that were admitted through the University of Chicago system between years 1992 to 2002. African samples were obtained through collaboration with O. Olopade and Nigerian resources, as described in Huo, 2009 [58].

Our study objectives included an assessment of CARM1 expression or localization and its associations with 11 clinical factors. Specifically, in our analyses cyt-CARM1 and nuc-CARM1 were the main response variables while the clinical factors were the potential explanatory variables. The clinical factors included; age and tumor size as continuous variables, scores (on a scale of 0–3) for tumor grade, ck5/6 IHC scores and EFGR IHC scores used as ordered scale variables, nuclear receptor status of ER, PR, HER2 as dichotomous 0/1 indicators and purely categorical variables for molecular tumor subtype (Luminal A, Luminal B, HER2+, Basal-like/Triple Negative) and histological tumor subtype (DCIS, Invasive, Metastatic). Lastly, we conducted a preliminary investigation on CARM1-survival associations. Of our cohort samples with complete CARM1, ER and PR data, 46% and 34% were scored positive for ER and PR, respectively. See statistical analyses section for more information on testing design. (See Additional file 3: Table S2 for specific samples’ details and distributions).

### Tissue microarray construction and composition

All samples used for this study were fixed in 10% formalin and embedded in paraffin. Representative areas of the different lesions (metastases into regional lymph nodes, invasive carcinomas, carcinomas in situ, adjacent normal epithelium) were carefully selected from hematoxylin and eosin stained sections and marked on individual paraffin blocks for the creation of TMA. The tissue cores 1mm in diameter were precisely arrayed into a new paraffin block as described by Kononen et al. [59]. TMAs were generated using an automated arrayer (ATA-27, Beecher, Inc., Sun Prairie, WI). A series of 15 TMAs were constructed, with a combined composition of 857 cores representing tumor and normal tissue specimens obtained from the Histology and Pathology Department of University of Chicago and from Nigeria, Africa, through collaboration between native hospitals and O. Olopade. In our TMA designs, we have included a sub-set of individuals with multiple cores. Each core was obtained from a distinct tumor and is not considered a ‘same-tumor’ replicate, but rather represents multiple lesions in the same individual at the time of breast cancer diagnosis and surgical resection. Of note, we surveyed the occurrence of multiple samples and observed that African women were more likely to have only 1 core (mean = 1.2 cores), relative to Caucasian or African-Americans (mean = 1.8 cores). However, this variation is most likely due to sample-gathering mechanisms and protocols that differ between African and USA study sites. For individuals with metastases samples (n = 50), we have collected matching primary site specimens and with the exception of 9 samples possibly lost during slide processing; these samples were paired and indicated in specified analyses.

### Molecular and histological assessment of tumor subtypes

The tumor subtypes were defined in two ways, histological subtypes and molecular subtypes. The histological subtypes are based upon histological characteristics of tumor growth and morphology upon visual microscopic evaluation by the certified histologist. The categories of the histological subtypes include: DCIS, Invasive, Metastatic or Normal. Tumors which did not fall under these subtypes were not included in histological subtype analyses and did not have a suitable number to establish additional categories (i.e. metaplastic). The molecular subtypes were determined based upon categorical molecular markers which are used to diagnose the clinical status of the disease. These markers include the expression of; ER, PR, EGFR, HER2 (gene amplification) and ck 5/6. The categories of the molecular subtype include; Luminal A, Luminal B, basal-like, HER2+, triple-negative and unclassified and were defined as previously reported [58]. In brief, these subtypes are defined based upon the expression levels of specific hormone receptors (Estrogen Receptor (ER), Progesterone Receptor (PR) and v-erb-b2 erythroblastic leukemia viral oncogene homolog 2 (ERBB2 or HER2). The presence of ER defines the Luminal subtypes and the absence of PR distinguishes Luminal B from Luminal B. The absence of all three is defined as “Triple Negative”. When the triple negative tumors are further characterized with EGFR and ck5/6 expression, they are then categorized as ‘Basal-like’. The uncategorized tumors lacked ER expression but data on other markers was not successfully obtained. Tumors where categorized into one of five categories for statistical analysis involving molecular subtypes, as the “triple-negative” tumors were combined with “unclassified”.

### Immunohistochemical staining and scoring

Primary antibodies against CARM1 were obtained from a commercial source (ABCam; ab110024). IHC conditions were optimized for proper dilution and secondary antibody colorimetric development by the University of Chicago Immunohistochemistry core facility using normal tissue whole section serial dilution series. (Specific protocol can be provided via personal communication.) Evaluation of immunohistochemical staining IHC scoring was performed by two independent reviewers without knowledge of patient outcomes. All discrepancies were resolved by a second examination by two observers simultaneously using a multi-head microscope. The semi-quantitative analysis was based on the evaluation of the intensity of cytoplasmic and nuclear reaction for CARM1 analogous to the scoring system as described by Hong H et al. [48].

Histological grading was performed based upon the Elston-Ellis modified Scarff-Bloom-Richardson method and a World Health Organization based three-tier grade [60-64]. The results of the Brown Staining were evaluated by visual inspection and based on the intensity of the stain using a standard scale of 0–3 and as previously reported for another subset of this population cohort ([58])Cut-off for positive staining was ≥10% of cells in tissue core area and was assessed for each individual sample of each TMA. A random subset of 50 samples were independently evaluated by one additional pathologist to confirm scoring ranges and consistency. Examples of negative control stains are in Additional file 4: Figure S2.

### Statistical analyses

For our statistical measures we focused on 11 factors, including; nuclear receptor status (ER and PR assessed as positive or negative), HER2 amplification status (assessed as positive or negative); IHC scores of ck5/6 and EFGR (scored on a 0–3 scale of intensity), molecular or histological tumor subtype (see Pathologic Assessment section of Methods), pathological grade (scored on a scale of 1–3), age at diagnosis and tumor size. We also conducted preliminary survival studies, which were separated into studies using only invasive cases and studies which were inclusive of all molecular and histological subtype category. Some secondary survival analyses were conducted within molecular categories, but small sample size renders these as preliminary observations.

Following discards from IHC processing damage and QC assessment, there was sufficient CARM1 data for 796 samples obtained from 549 individuals, with 79 Cau, 92 Afr. Am. and 239 Africans (Additional file 3: Table S2). IHC processing of other factors measured resulted in artifacts and the additional loss of certain samples and therefore are missing data necessary for some specific statistical comparison. These were discarded for those particular analyses which the missing data are relevant. The number of samples used for each major analysis is outlined in Table 1. Specifically, tumor subtypes were considered in two ways, histological and molecular. The ‘histological subtype’ parameter includes the sorting of DCIS, invasive and metastatic categories, while the ‘molecular subtype’ parameter includes the sorting of “Luminal A”, “Luminal B”, “HER2+”, “unclassified ER-“ and “basal-like”, based upon prior IHC analysis of these samples and cases [65,66]. Subsequent analyses for patho-clinical associations and overall survival outcomes were initially based solely on invasive cases for which only 371 patients were used. In all statistical tests, P-values less than 0.05 were considered significant.

The primary response variables are cyt-CARM1 and nuc-CARM1. The CARM1 scores were obtained in the same manner as the EGFR and ck5/6 scores, on a 0–3 scale. Because the expression levels of nuc-CARM1 are not directly correlated with cyt-CARM1 levels, we measured the associations of these variables separately and also created a “relative CARM1 score” that equates to the nuclear score, subtracted from the cytoplasmic score.

ANOVA and/or Random-effects ordinal regression models were used for comparisons among tumor types. Kruskal-Wallis tests were used determine significant differences among variables. Spearman correlations were used to determine relatedness. The Wilcoxon rank-sum test was used in ethnicity tests and Kaplan-Meier generated survival curves underwent log-rank tests for survival associations. All statistical analyses were performed and validated by independent statistician consultants using various SAS and STATA 9.0 statistical packages as previously described [58].

Search Tool for the Retrieval of Interacting Genes/Proteins (STRING) version 9.0 was used to determine the interaction model.

## Abbreviations

ER: Estrogen receptor; PR: Progesterone receptor; CARM1: (Co-Activator Arginine Methyltransferase); EGFR: Epidermal growth factor receptor; HER2/ERBB2: Human epidermal growth receptor 2; Afr: African; Afr.Am.: African American; Cau: Caucasian; cyt: cytoplasmic; nuc: nuclear; ANOVA: Analysis of variance; ck: cytokeratin; IHC: Immunohistochemical; TMA: Tissue microarray; SNP: Single nucleotide polymorphism; AIM: Ancestral informative marker; WSR: Wilcoxon signed rank; P: p-value; LN: Lymph node.

## Competing interests

There are no competing interests to disclose.

## Authors’ contributions

MBD conceived the study and coordinated the acquisition of TMA samples and data, including; optimization of IHC protocol for use in the U of C facility and interpretations of statistical analyses OO is the senior author and advisor on experimental design and tissue use. DH compiled initial scores for, and conducted statistical analyses. AK performed the patho-histo scoring for the TMA samples and designed and produced the TMAs. All statistical findings were independently validated by the University of Georgia Statistics Service Center, by XL and SW under the direction of JR. All authors have read and approved the final manuscript.

## Supplementary Material

Additional file 1: Table S1 The summary of numbers of patients with indicated tissue/tumor types. A subset of these are used for our intra-patient analyses. The patients analyzed depends on the presence of the tumor types in the comparison and successful stains for both nuc-CARM1 and cyt-CARM1 for all samples for that patient. The actual number of patients utilized for each test is indicated in Table 3. Click here for file

Additional file 2: Figure S1 Kaplan-Meier curves for cyt-CARM1 within the ER negative (top) and ER positive (bottom) tumor categories. We find a greatly significant association with survival and cyt-CARM1 for ER negative cases, but no association within the ER positive cases. Click here for file

Additional file 3: Table S2 Descriptive Tables of samples. Of the samples that were retained for statistical analysis, the distributions within categorical groupings are listed in indicated tables. These numbers are reduced from the original numbers included in the TMA design, if specific core samples were lost or damaged during IHC processing of any category. Click here for file

Additional file 4: Figure S2 Representative secondary antibody control for our CARM1 IHC protocols. Click here for file

## References

[B1] Curtis E Quale C Haggstrom D Smith-Bindman R Racial and ethnic differences in breast cancer survival: how much is explained by screening, tumor severity, biology, treatment, comorbidities, and demographics? Cancer 2008 112 1 171 180 10.1002/cncr.23131 18040998 PMC2674622

[B2] Vainshtein J Disparities in breast cancer incidence across racial/ethnic strata and socioeconomic status: a systematic review J Natl Med Assoc 2008 100 7 833 839 18672561 10.1016/s0027-9684(15)31378-x

[B3] Bowen RL Duffy SW Ryan DA Hart IR Jones JL Early onset of breast cancer in a group of British black women Br J Cancer 2008 98 2 277 281 10.1038/sj.bjc.6604174 18182985 PMC2361454

[B4] Blackman DJ Masi CM Racial and ethnic disparities in breast cancer mortality: are we doing enough to address the root causes? J Clin Oncol 2006 24 14 2170 2178 10.1200/JCO.2005.05.4734 16682736

[B5] Fejerman L Ziv E Population differences in breast cancer severity Pharmacogenomics 2008 9 3 323 333 10.2217/14622416.9.3.323 18303968

[B6] Baquet CR Mishra SI Commiskey P Ellison GL DeShields M Breast cancer epidemiology in blacks and whites: disparities in incidence, mortality, survival rates and histology J Natl Med Assoc 2008 100 5 480 488 18507200 10.1016/s0027-9684(15)31294-3 PMC3603001

[B7] Benn-Torres J Bonilla C Robbins CM Waterman L Moses TY Hernandez W Santos ER Bennett F Aiken W Tullock T Coard K Hennis A Wu S Nemesure B Leske MC Freeman V Carpten J Kittles RA Admixture and population stratification in African Caribbean populations Ann Hum Genet 2008 72 Pt 1 90 98 17908263 10.1111/j.1469-1809.2007.00398.x

[B8] Jovanovic J Ronneberg JA Tost J Kristensen V The epigenetics of breast cancer Mol Oncol 2010 4 3 242 254 10.1016/j.molonc.2010.04.002 20627830 PMC5527941

[B9] Hon GC Hawkins RD Caballero OL Lo C Lister R Pelizzola M Valsesia A Ye Z Kuan S Edsall LE Camargo AA Stevenson BJ Ecker JR Bafna V Strausberg RL Simpson AJ Ren B Global DNA hypomethylation coupled to repressive chromatin domain formation and gene silencing in breast cancer Genome Res 2011 10.1101/gr.125872.111 PMC3266032 22156296

[B10] Hinshelwood RA Huschtscha LI Melki J Stirzaker C Abdipranoto A Vissel B Ravasi T Wells CA Hume DA Reddel RR Clark SJ Concordant epigenetic silencing of transforming growth factor-beta signaling pathway genes occurs early in breast carcinogenesis Cancer Res 2007 67 24 11517 11527 10.1158/0008-5472.CAN-07-1284 18089780

[B11] Chernov AV Baranovskaya S Golubkov VS Wakeman DR Snyder EY Williams R Strongin AY Microarray-based transcriptional and epigenetic profiling of matrix metalloproteinases, collagens, and related genes in cancer J Biol Chem 2010 285 25 19647 19659 10.1074/jbc.M109.088153 20404328 PMC2885243

[B12] Bediaga NG Acha-Sagredo A Guerra I Viguri A Albaina C Ruiz Diaz I Rezola R Alberdi MJ Dopazo J Montaner D de Renobales M Fernandez AF Field JK Fraga MF Liloglou T de Pancorbo MM DNA methylation epigenotypes in breast cancer molecular subtypes Breast cancer research: BCR 2010 12 5 R77 10.1186/bcr2721 20920229 PMC3096970

[B13] Veeck J Esteller M Breast cancer epigenetics: from DNA methylation to microRNAs J Mammary Gland Biol Neoplasia 2010 15 1 5 17 10.1007/s10911-010-9165-1 20101446 PMC2824126

[B14] Lavasani MA Moinfar F Molecular classification of breast carcinomas with particular emphasis on "basal-like" carcinoma: A critical review J Biophotonics 2012 10.1002/jbio.201100097 22232077

[B15] Pazaiti A Fentiman IS Basal phenotype breast cancer: implications for treatment and prognosis Womens Health (Lond Engl) 2011 7 2 181 202 10.2217/whe.11.5 21410345

[B16] Gusterson BA Ross DT Heath VJ Stein T Basal cytokeratins and their relationship to the cellular origin and functional classification of breast cancer Breast Cancer Res 2005 7 4 143 148 10.1186/bcr1041 15987465 PMC1175069

[B17] Fulford LG Easton DF Reis-Filho JS Sofronis A Gillett CE Lakhani SR Hanby A Specific morphological features predictive for the basal phenotype in grade 3 invasive ductal carcinoma of breast Histopathology 2006 49 1 22 34 10.1111/j.1365-2559.2006.02453.x 16842243

[B18] Dalvai M Bystricky K The role of histone modifications and variants in regulating gene expression in breast cancer J Mammary Gland Biol Neoplasia 2010 15 1 19 33 10.1007/s10911-010-9167-z 20131086

[B19] Jin W Chen L Chen Y Xu SG Di GH Yin WJ Wu J Shao ZM UHRF1 is associated with epigenetic silencing of BRCA1 in sporadic breast cancer Breast Cancer Res Treat 2010 123 2 359 373 10.1007/s10549-009-0652-2 19943104

[B20] Xu H Wei CL Lin F Sung WK An HMM approach to genome-wide identification of differential histone modification sites from ChIP-seq data Bioinformatics 2008 24 20 2344 2349 Epub 2008 Jul 29 10.1093/bioinformatics/btn402 18667444

[B21] Zika E Fauquier L Vandel L Ting JP Interplay among coactivator-associated arginine methyltransferase 1, CBP, and CIITA in IFN-gamma-inducible MHC-II gene expression Proc Natl Acad Sci U S A 2005 102 45 16321 16326 10.1073/pnas.0505045102 16254053 PMC1283426

[B22] Barr FD Krohmer LJ Hamilton JW Sheldon LA Disruption of histone modification and CARM1 recruitment by arsenic represses transcription at glucocorticoid receptor-regulated promoters PLoS One 2009 4 8 e6766 10.1371/journal.pone.0006766 19707557 PMC2727952

[B23] Daujat S Bauer UM Shah V Turner B Berger S Kouzarides T Crosstalk between CARM1 methylation and CBP acetylation on histone H3 Current biology: CB 2002 12 24 2090 2097 10.1016/S0960-9822(02)01387-8 12498683

[B24] Chernov AV Sounni NE Remacle AG Strongin AY Epigenetic control of the invasion-promoting MT1-MMP/MMP-2/TIMP-2 axis in cancer cells J Biol Chem 2009 284 19 12727 12734 10.1074/jbc.M900273200 19286653 PMC2676002

[B25] Lee DY Northrop JP Kuo MH Stallcup MR Histone H3 lysine 9 methyltransferase G9a is a transcriptional coactivator for nuclear receptors J Biol Chem 2006 281 13 8476 8485 10.1074/jbc.M511093200 16461774 PMC1770944

[B26] Lim S Janzer A Becker A Zimmer A Schule R Buettner R Kirfel J Lysine-specific demethylase 1 (LSD1) is highly expressed in ER-negative breast cancers and a biomarker predicting aggressive biology Carcinogenesis 2010 31 3 512 520 10.1093/carcin/bgp324 20042638

[B27] Lu TY Kao CF Lin CT Huang DY Chiu CY Huang YS Wu HC DNA methylation and histone modification regulate silencing of OPG during tumor progression J Cell Biochem 2009 108 1 315 325 10.1002/jcb.22256 19565568

[B28] Kim YR Lee BK Park RY Nguyen NT Bae JA Kwon DD Jung C Differential CARM1 expression in prostate and colorectal cancers BMC cancer 2010 10 197 10.1186/1471-2407-10-197 20462455 PMC2881889

[B29] Xu W Chen H Du K Asahara H Tini M Emerson BM Montminy M Evans RM A transcriptional switch mediated by cofactor methylation Science 2001 294 5551 2507 2511 10.1126/science.1065961 11701890

[B30] Majumder S Liu Y Ford OH 3rd Mohler JL Whang YE Involvement of arginine methyltransferase CARM1 in androgen receptor function and prostate cancer cell viability Prostate 2006 66 12 1292 1301 10.1002/pros.20438 16705743

[B31] Kovalchuk O Tryndyak VP Montgomery B Boyko A Kutanzi K Zemp F Warbritton AR Latendresse JR Kovalchuk I Beland FA Pogribny IP Estrogen-induced rat breast carcinogenesis is characterized by alterations in DNA methylation, histone modifications and aberrant microRNA expression Cell Cycle 2007 6 16 2010 2018 10.4161/cc.6.16.4549 17700064

[B32] da Costa Prando E Cavalli LR Rainho C Evidence of epigenetic regulation of the tumor suppressor gene cluster flanking RASSF1 in breast cancer cell lines Epigenetics 2011 6 12 1413 1424 10.4161/epi.6.12.18271 22139571 PMC3256331

[B33] Chen D Ma H Hong H Koh SS Huang SM Schurter BT Aswad DW Stallcup MR Regulation of transcription by a protein methyltransferase Science 1999 284 5423 2174 2177 10.1126/science.284.5423.2174 10381882

[B34] Frietze S Lupien M Silver PA Brown M CARM1 regulates estrogen-stimulated breast cancer growth through up-regulation of E2F1 Cancer Res 2008 68 1 301 306 10.1158/0008-5472.CAN-07-1983 18172323

[B35] El Messaoudi S Fabbrizio E Rodriguez C Chuchana P Fauquier L Cheng D Theillet C Vandel L Bedford MT Sardet C Coactivator-associated arginine methyltransferase 1 (CARM1) is a positive regulator of the Cyclin E1 gene Proc Natl Acad Sci U S A 2006 103 36 13351 13356 10.1073/pnas.0605692103 16938873 PMC1569167

[B36] Feng Q Yi P Wong J O'Malley BW Signaling within a coactivator complex: methylation of SRC-3/AIB1 is a molecular switch for complex disassembly Mol Cell Biol 2006 26 21 7846 7857 10.1128/MCB.00568-06 16923966 PMC1636757

[B37] Shirley SH Rundhaug JE Tian J Cullinan-Ammann N Lambertz I Conti CJ Fuchs-Young R Transcriptional regulation of estrogen receptor-alpha by p53 in human breast cancer cells Cancer Res 2009 69 8 3405 3414 10.1158/0008-5472.CAN-08-3628 19351845 PMC3079369

[B38] Ceschin DG Walia M Wenk SS Duboe C Gaudon C Xiao Y Fauquier L Sankar M Vandel L Gronemeyer H Methylation specifies distinct estrogen-induced binding site repertoires of CBP to chromatin Genes Dev 2011 25 11 1132 1146 10.1101/gad.619211 21632823 PMC3110952

[B39] Al-Dhaheri M Wu J Skliris GP Li J Higashimato K Wang Y White KP Lambert P Zhu Y Murphy L Xu W CARM1 is an important determinant of ERalpha-dependent breast cancer cell differentiation and proliferation in breast cancer cells Cancer Res 2011 71 6 2118 2128 10.1158/0008-5472.CAN-10-2426 21282336 PMC3076802

[B40] Choi YL Oh E Park S Kim Y Park YH Song K Cho EY Hong YC Choi JS Lee JE Kim JH Nam SJ Im YH Yang JH Shin YK Triple-negative, basal-like, and quintuple-negative breast cancers: better prediction model for survival BMC Cancer 2010 10 507 10.1186/1471-2407-10-507 20860845 PMC2957395

[B41] Clark SE Warwick J Carpenter R Bowen RL Duffy SW Jones JL Molecular subtyping of DCIS: heterogeneity of breast cancer reflected in pre-invasive disease Br J Cancer 2011 104 1 120 127 10.1038/sj.bjc.6606021 21139586 PMC3039794

[B42] De Laurentiis M Cianniello D Caputo R Stanzione B Arpino G Cinieri S Lorusso V De Placido S Treatment of triple negative breast cancer (TNBC): current options and future perspectives Cancer Treat Rev 2010 36 Suppl 3 S80 86 10.1016/s0305-7372(10)70025-6 21129616

[B43] Ishihara A Tsuda H Kitagawa K Yoneda M Shiraishi T Morphological characteristics of basal-like subtype of breast carcinoma with special reference to cytopathological features Breast Cancer 2009 16 3 179 185 10.1007/s12282-009-0108-x 19466513

[B44] Kao J Salari K Bocanegra M Choi YL Girard L Gandhi J Kwei KA Hernandez-Boussard T Wang P Gazdar AF Minna JD Pollack JR Molecular profiling of breast cancer cell lines defines relevant tumor models and provides a resource for cancer gene discovery PLoS One 2009 4 7 e6146 10.1371/journal.pone.0006146 19582160 PMC2702084

[B45] Cheng D Bedford MT Xenoestrogens regulate the activity of arginine methyltransferases Chembiochem: a European Journal of Chemical Biology 2011 12 2 323 329 10.1002/cbic.201000522 21243720 PMC3142315

[B46] Carascossa S Dudek P Cenni B Briand PA Picard D CARM1 mediates the ligand-independent and tamoxifen-resistant activation of the estrogen receptor alpha by cAMP Genes Dev 2010 24 7 708 719 10.1101/gad.568410 20360387 PMC2849127

[B47] Baek SH Ohgi KA Nelson CA Welsbie D Chen C Sawyers CL Rose DW Rosenfeld MG Ligand-specific allosteric regulation of coactivator functions of androgen receptor in prostate cancer cells Proc Natl Acad Sci U S A 2006 103 9 3100 3105 10.1073/pnas.0510842103 16492776 PMC1413901

[B48] Hong H Kao C Jeng MH Eble JN Koch MO Gardner TA Zhang S Li L Pan CX Hu Z MacLennan GT Cheng L Aberrant expression of CARM1, a transcriptional coactivator of androgen receptor, in the development of prostate carcinoma and androgen-independent status Cancer 2004 101 1 83 89 10.1002/cncr.20327 15221992

[B49] Szklarczyk D Franceschini A Kuhn M Simonovic M Roth A Minguez P Doerks T Stark M Muller J Bork P Jensen LJ von Mering C The STRING database in 2011: functional interaction networks of proteins, globally integrated and scored Nucleic Acids Res 2011 39 Database issue D561 568 10.1093/nar/gkq973 21045058 PMC3013807

[B50] Fujiwara T Mori Y Chu DL Koyama Y Miyata S Tanaka H Yachi K Kubo T Yoshikawa H Tohyama M CARM1 regulates proliferation of PC12 cells by methylating HuD Mol Cell Biol 2006 26 6 2273 2285 10.1128/MCB.26.6.2273-2285.2006 16508003 PMC1430293

[B51] Hsu JM Chen CT Chou CK Kuo HP Li LY Lin CY Lee HJ Wang YN Liu M Liao HW Shi B Lai CC Bedford MT Tsai CH Hung MC Crosstalk between Arg 1175 methylation and Tyr 1173 phosphorylation negatively modulates EGFR-mediated ERK activation Nature Cell Biology 2011 13 2 174 181 10.1038/ncb2158 21258366 PMC3048027

[B52] Agurs-Collins T Dunn BK Browne D Johnson KA Lubet R Epidemiology of health disparities in relation to the biology of estrogen receptor-negative breast cancer Semin Oncol 2010 37 4 384 401 10.1053/j.seminoncol.2010.05.002 20816508

[B53] Morgan DK Whitelaw E The case for transgenerational epigenetic inheritance in humans Mamm Genome 2008 19 6 394 397 Epub 2008 Jul 29. Review. 10.1007/s00335-008-9124-y 18663528

[B54] Miljkovic-Gacic I Ferrell RE Patrick AL Kammerer CM Bunker CH Estimates of African, European and Native American ancestry in Afro-Caribbean men on the island of Tobago Hum Hered 2005 60 3 129 133 10.1159/000089553 16282694

[B55] Reiner AP Carlson CS Ziv E Iribarren C Jaquish CE Nickerson DA Genetic ancestry, population sub-structure, and cardiovascular disease-related traits among African-American participants in the CARDIA study Hum Genet 2007 121 5 565 575 10.1007/s00439-007-0350-2 17356887

[B56] Ruiz-Narvaez EA Rosenberg L Rotimi CN Cupples LA Boggs DA Adeyemo A Cozier YC Adams-Campbell LL Palmer JR Genetic variants on chromosome 5p12 are associated with risk of breast cancer in African American women: the Black Women's Health Study Breast cancer research and treatment 2010 123 2 525 530 10.1007/s10549-010-0775-5 20140701 PMC3208423

[B57] Rybicki BA Levin AM McKeigue P Datta I Gray-McGuire C Colombo M Reich D Burke RR Iannuzzi MC A genome-wide admixture scan for ancestry-linked genes predisposing to sarcoidosis in African-Americans Genes and immunity 2011 12 2 67 77 10.1038/gene.2010.56 21179114 PMC3058725

[B58] Huo D Ikpatt F Khramtsov A Dangou JM Nanda R Dignam J Zhang B Grushko T Zhang C Oluwasola O Malaka D Malami S Odetunde A Adeoye AO Iyare F Falusi A Perou CM Olopade OI Population differences in breast cancer: survey in indigenous African women reveals over-representation of triple-negative breast cancer J Clin Oncol 2009 27 27 4515 4521 10.1200/JCO.2008.19.6873 19704069 PMC2754904

[B59] Kononen J Bubendorf L Kallioniemi A Barlund M Schraml P Leighton S Torhorst J Mihatsch MJ Sauter G Kallioniemi OP Tissue microarrays for high-throughput molecular profiling of tumor specimens Nature medicine 1998 4 7 844 847 10.1038/nm0798-844 9662379

[B60] Elston EW Ellis IO Method for grading breast cancer J Clin Pathol 1993 46 2 189 190 8459046 10.1136/jcp.46.2.189-b PMC501162

[B61] Albert R Muller JG Kristen P Schindewolf T Kneitz S Harms H New method of nuclear grading of tissue sections by means of digital image analysis with prognostic significance for node-negative breast cancer patients Cytometry 1996 24 2 140 150 10.1002/(SICI)1097-0320(19960601)24:2<140::AID-CYTO6>3.0.CO;2-N 8725663

[B62] Tawfik OW Kimler BF Davis M Donahue JK Persons DL Fan F Hagemeister S Thomas P Connor C Jewell W Fabian CJ Comparison of immunohistochemistry by automated cellular imaging system (ACIS) versus fluorescence in-situ hybridization in the evaluation of HER-2/neu expression in primary breast carcinoma Histopathology 2006 48 3 258 267 10.1111/j.1365-2559.2005.02322.x 16430472

[B63] Tawfik O Kimler BF Davis M Stasik C Lai SM Mayo MS Fan F Donahue JK Damjanov I Thomas P Connor C Jewell WR Smith H Fabian CJ Grading invasive ductal carcinoma of the breast: advantages of using automated proliferation index instead of mitotic count Virchows Archiv: an international journal of pathology 2007 450 6 627 636 10.1007/s00428-007-0400-0 17458558

[B64] Tawfik O Davis K Kimler BF Davis MK Hull S Fan F Khan QJ O'Dea AP Thomas P Clinicopathological characteristics of triple-negative invasive mammary carcinomas in African-American versus Caucasian women Ann Clin Lab Sci 2010 40 4 315 323 20947804

[B65] Khramtsov AI Khramtsova GF Tretiakova M Huo D Olopade OI Goss KH Wnt/beta-catenin pathway activation is enriched in basal-like breast cancers and predicts poor outcome Am J Pathol 2010 176 6 2911 2920 10.2353/ajpath.2010.091125 20395444 PMC2877852

[B66] Huo D Adebamowo CA Ogundiran TO Akang EE Campbell O Adenipekun A Cummings S Fackenthal J Ademuyiwa F Ahsan H Olopade OI Parity and breastfeeding are protective against breast cancer in Nigerian women Br J Cancer 2008 98 5 992 996 10.1038/sj.bjc.6604275 18301401 PMC2266848

