# Supplementary material for: Expression and sub-cellular localization of an epigenetic regulator, co-activator arginine methyltransferase 1 (CARM1), is associated with specific breast cancer subtypes and ethnicity
Source: Mol Cancer. 2013 May 10;12:40. doi: 10.1186/1476-4598-12-40 (PMC3663705; doi:10.1186/1476-4598-12-40)
Supplement: Additional file 2: Figure S1 — Kaplan-Meier curves for cyt-CARM1 within the ER negative (top) and ER positive (bottom) tumor categories. We find a greatly significant association with survival and cyt-CARM1 for ER negative cases, but no association within the ER positive cases. [file 1476-4598-12-40-S2.pdf]

# Supp Fig 1. Cyto-CARM1 survival associations by ER status

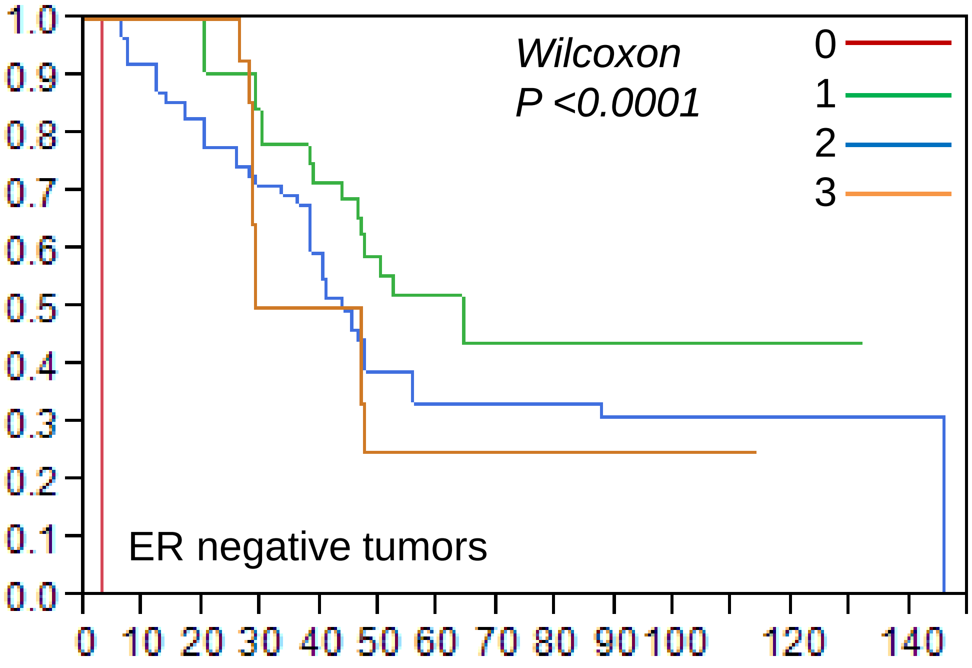


| Group | Number failed | Number censored | Mean | | Std Error |
|---|---|---|---|---|---|
| 0 | 1 | 0 | 2.98973 | | . |
| 1 | 17 | 18 | 50.6772 | Biased | 2.87251 |
| 2 | 43 | 23 | 68.5101 | | 7.143 |
| 3 | 10 | 4 | 37.579 | Biased | 2.71505 |
| Combined | 71 | 45 | 72.4725 | | 5.38971 |

**Quantiles**

| Group | Median Time | Lower 95% | Upper 95% | 25% Failures | 75% Failures |
|---|---|---|---|---|---|
| 0 | 2.9897 | . | . | 2.9897 | 2.9897 |
| 1 | 63.869 | 43.63 | . | 38.259 | . |
| 2 | 43.63 | 37.947 | 55.622 | 25.561 | 145.84 |
| 3 | 37.848 | 27.992 | 47.474 | 27.992 | . |
| Combined | 46.62 | 39.951 | 55.622 | 28.945 | 145.84 |

**Tests Between Groups**

| Test | ChiSquare | DF | Prob>ChiSq |
|---|---|---|---|
| Log-Rank | 114.2507 | 3 | <.0001* |
| Wilcoxon | 115.0641 | 3 | <.0001* |

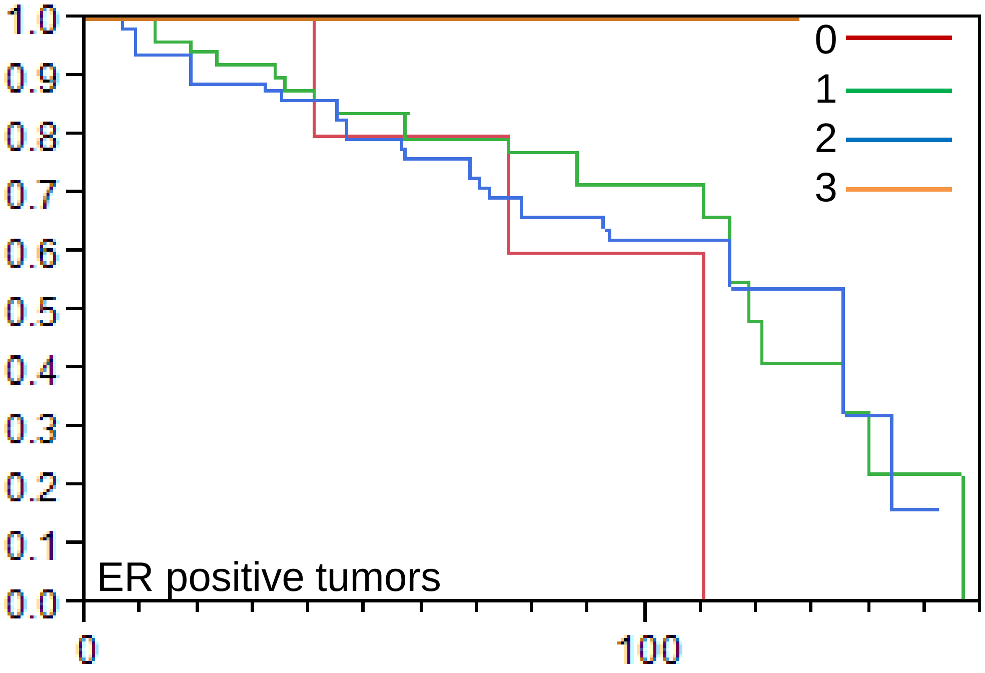


| Group | Number failed | Number censored | Mean | | Std Error |
|---|---|---|---|---|---|
| 0 | 3 | 2 | 89.2518 | | 15.3084 |
| 1 | 22 | 30 | 109.764 | | 7.26686 |
| 2 | 29 | 40 | 103.376 | Biased | 6.18918 |
| 3 | 0 | 5 | . | | . |
| Combined | 54 | 77 | 109.233 | | 4.70401 |

**Quantiles**

| Group | Median Time | Lower 95% | Upper 95% | 25% Failures | 75% Failures |
|---|---|---|---|---|---|
| 0 | 110.23 | 40.378 | 110.23 | 75.203 | 110.23 |
| 1 | 117.98 | 110.23 | 139.47 | 87.819 | 139.47 |
| 2 | 135.13 | 93.306 | 143.84 | 68.37 | 143.84 |
| 3 | . | . | . | . | . |
| Combined | 135.13 | 115.02 | 139.47 | 75.203 | 143.84 |

**Tests Between Groups**

| Test | ChiSquare | DF | Prob>ChiSq |
|---|---|---|---|
| Log-Rank | 3.5345 | 3 | 0.3163 |
| Wilcoxon | 2.7991 | 3 | 0.4237 |
