# Supplementary figures and images for: Expression and sub-cellular localization of an epigenetic regulator, co-activator arginine methyltransferase 1 (CARM1), is associated with specific breast cancer subtypes and ethnicity
Source: Mol Cancer. 2013 May 10;12:40. doi: 10.1186/1476-4598-12-40 (PMC3663705; doi:10.1186/1476-4598-12-40)

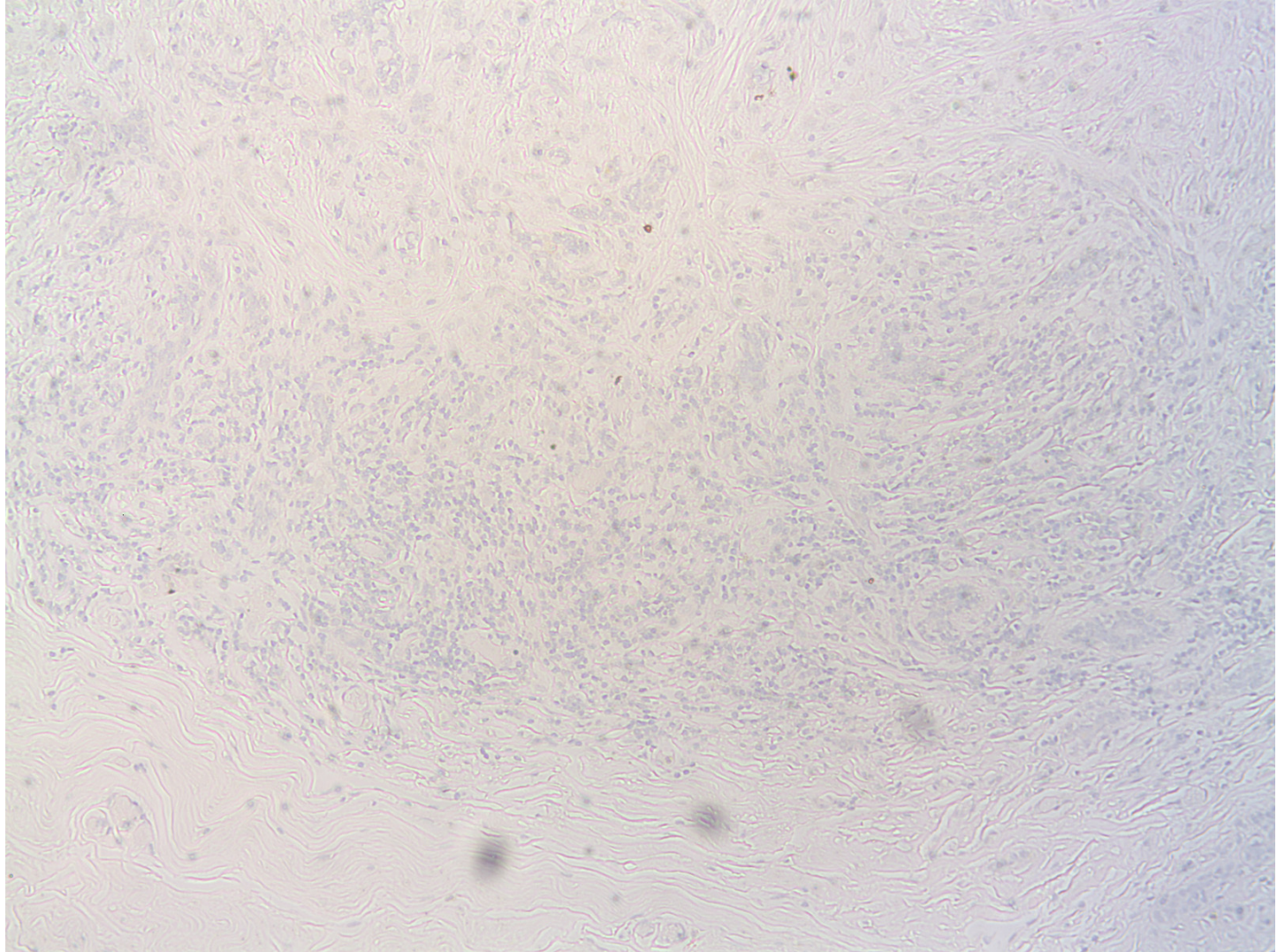

Supplement: Additional file 4: Figure S2 — Representative secondary antibody control for our CARM1 IHC protocols. [file 1476-4598-12-40-S4.pdf]
